# Supplementary material for: Neutrophil-specific STAT4 deficiency attenuates atherosclerotic burden and improves plaque stability via reduction in neutrophil activation and recruitment into aortas of Ldlr−/− mice
Source: Front Cardiovasc Med. 2023 Jun 16;10:1175673. doi: 10.3389/fcvm.2023.1175673 (PMC10313069; doi:10.3389/fcvm.2023.1175673)
Supplement: Supplementary file 1 [file Presentation1.pdf]

Supplemental Table 1. List of all antibodies, dilutions, and vendor sources.

Marker	Fluor	Clone	Vendor	Dilution
Lineage	Percp	*	Biolegend	1/400
Sca-1	PE-Cy7	D7	Thermo Fisher	1/400
c-Kit	APC	2B8	Thermo Fisher	1/400
FcγR1	AF-647	X54-5/7.1	BD Biosciences	1/400
CD48	APC e780	HM48-1	Thermo Fisher	1/400
CD135	BV 421	A2F10	Biolegend	1/400
CD11b	eFluor 450	M1/70	Thermo Fisher	1/800
Ly6G	PerCP-eFluor 710	1A8	Thermo Fisher	1/800
CCR1	APC	643854	R&D Systems	1/400
CCR2	FITC	475301	R&D Systems	1/400
MitoSOX Red	N/A	N/A	Thermo Fisher	100 nM
CD63	PE-Cy7	H5C6	Thermo Fisher	1/400
CD41	BV-605	MWReg30	BD Biosciences	1/1600
CD45	PerCP-Cy5.5	30-F11	Biolegend	1/400
α-SMA	Alexa Fluor 647	D4K9N	Cell Signaling Technology	1/200
*Lin was comprised of a cocktail of CD4, CD8, CD11b, CD45R/B220, GR-1, CD11c, and Ter119 antibodies				

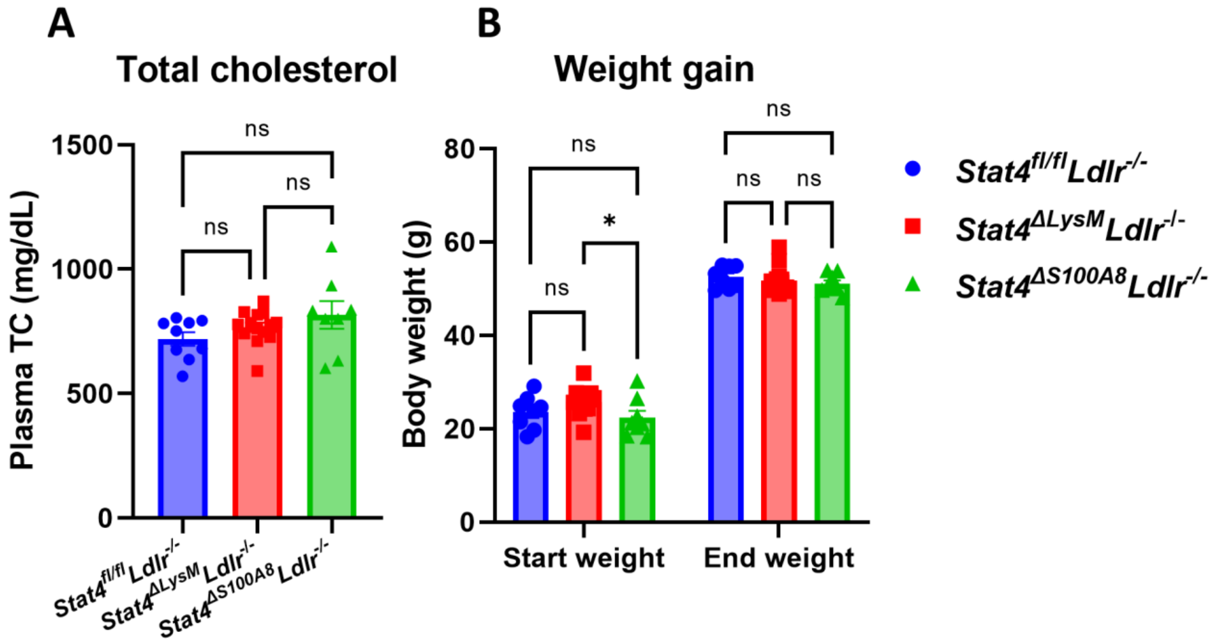

Supplemental Figure 1. (A) Total plasma cholesterol measurements at 28 weeks HFD-C feeding. (B) Beginning and final body weight measurements at 0 and 28 weeks HFD feeding. Data represent mean±SEM. One-way ANOVA (A) and Two-way ANOVA (B) with Tukey's multiple comparisons test. *$p<0.05$.

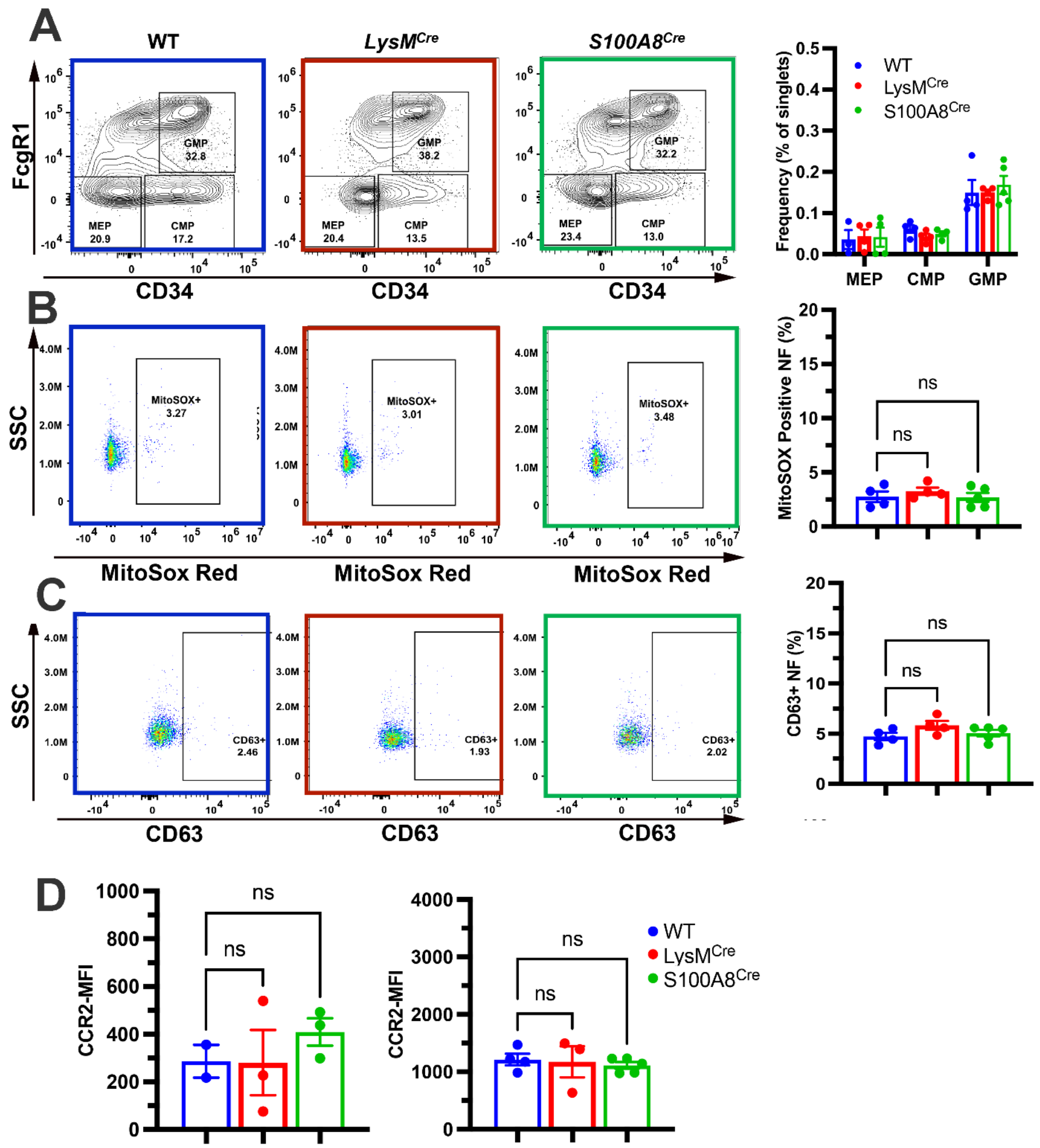

Supplemental Figure 2. Representative FACS plots and quantification for identifying granulocyte-macrophage progenitor (GMP), common myeloid progenitor (CMP), and megakaryocyte-erythrocyte progenitor (MEP) populations from Lin-Sca1-cKit+ bone marrow cells of chow diet fed C57BL/6, *LysM*^{cre},and *S100A8*^{cre} mice. (B) Representative FACS plots and quantification of basal mitochondrial superoxide production by MitoSOX Red positive stained blood neutrophils from of C57BL/6, *LysM*^{cre}*,* and *S100A8*^{cre} mice. (C) Representative FACS plot ans quantification of surface expression of degranulation marker CD63 on peripheral blood neutrophils from of C57BL/6, *LysM*^{cre}*,* and *S100A8*^{cre} mice. (D) Quantification of mean fluorescence intensity (MFI) for CCR1 and CCR2 expression on peripheral blood neutrophils from chow diet fed C57BL/6, *LysM*^{cre}, and *S100A8*^{cre} mice. Data represent mean±SEM. Unpaired Student's t test. n=4-5 mice/group. 2 independent experiments.
